# Dr. Yeates on the Treatment of Pulmonary Complaints

**Published:** 1799-03

**Authors:** G. D. Yeates

**Affiliations:** Bedford


SUUM CUIQUE.

SIR,

AS medical disquisitions will properly enter into the plan of your proposed journal, I hope the following observations will not be deemed irrelative to its contents. I have already, in my observations on modern discoveries, endeavoured to restore to the authors of the last century their long-lost honours; and I am happy in having an opportunity of not suffering the well-earned distinction of another to be eclipsed for want of due investigation. In a work* published about a year ago, a method, or a new principle, as is imagined, is introduced for the treatment of pulmonary complaints. "This principle," says the author, (which I conceive the reader will find established in the following remarks) "is that of a *particular and strict limitation* of liquids, during the treatment of every pulmonary disease. It appears wonderful that practitioners have *never thought* of this principle, but have, on the contrary, constantly treated patients labouring under pulmonary affections in the same way as if they had laboured under similar diseases of any other parts of the body." † Catarrh is a complaint which affects the lungs more or less in different persons, and often terminates in lingering phthisis, or in direct hæmoptysis; in the former, when the scrophulous influence is present, it forms tubercles; as well as in the latter, when the tender vessels in a delicate constitution are ruptured by repeated fits of coughing. In this point of view, catarrh may be termed a pulmonary disease in the strict sense of the word.—The idea of a limited use of liquids has been long ago taken up, and the practice of it subjected to experience.—"In quantum, igitur, 'says LOWER,' ex sero sanguinis materia catarrho suppeditatur, quicquid ei pabulum detrahit, aut serum per renes præcipitat, vel per alvum derivat, vel per poros corporis dispellit, intentioni curativæ apprime satisfacit. Quare quum catarrhus primo urget, nihil magis ei supprimendo conducit, modo absque febre sit, quam ut *sitim diutissime toleremus* tridui enim vel quatridui *abstinentia a potu* plures novi a catarrho prorsus liberatos, cujus alia ratio non est, quam quod fomite subducto omnino exsciccatur, non aliter ac rivuli exarescunt ex pluviarum penuria." (*De Catarrhis.*)

The

* Observations on the Pulmonary System, &c. &c. by WILLIAM DAVIDSON.

† Introduction, p. 9.

The intention of Mr. DAVIDSON, in putting his theory in practice, is to obviate the ftimulus of diftention; and that of LOWER, to diminifh the pabulum which fupplies the increafed fecretion in catarrh. Which is the better theory? Now that the humoral pathology is exploded, the theory of Lower muft give way to the action of veffels; but his obfervations and confequent practice have neverthelefs a claim to confiderable ingenuity.

Dr. DUNCAN, of Edinburgh, has frequently treated with fuccefs fome cafes of chronic catarrh, on another principle, mentioned by Lower, viz. by determining to the kidnies.

The Profeffor has given what in his opinion may be properly called a purgative diuretic, which, by leffening the afflux of fluids upon the lungs, diminifhes irritation, and cures, if I may be allowed the expreffion, by derivation and revulfion. The reafoning in Lower is precifely fimilar.

I am, Sir,

Your's, &c.

*Bedford, January* 9, 1799. G. D. YEATES.

---

### *A fingular Variety of the Difeafe termed Chorea Sancti Viti: Communicated by* G. C. DELARIVE, *M. D. Affiftant Phyfician to the Public Difpenfary, London.*

*To the Editor of the Medical and Phyfical Journal.*

